# An inverse method to determine the mechanical properties of the iris in vivo

**DOI:** 10.1186/1475-925X-13-66

**Published:** 2014-05-30

**Authors:** Kunya Zhang, Xiuqing Qian, Xi Mei, Zhicheng Liu

**Affiliations:** 1 School of Biomedical Engineering, Capital Medical University, Beijing 100069, China

**Keywords:** Iris, In vivo experiment, Finite element method, Multi-island genetic algorithm

## Abstract

**Background:**

Understanding the mechanical properties of the iris can help to have an insight into the eye diseases with abnormalities of the iris morphology. Material parameters of the iris were simply calculated relying on the ex vivo experiment. However, the mechanical response of the iris in vivo is different from that ex vivo, therefore, a method was put forward to determine the material parameters of the iris using the optimization method in combination with the finite element method based on the in vivo experiment.

**Material and methods:**

Ocular hypertension was induced by rapid perfusion to the anterior chamber, during perfusion intraocular pressures in the anterior and posterior chamber were record by sensors, images of the anterior segment were captured by the ultrasonic system. The displacement of the characteristic points on the surface of the iris was calculated. A finite element model of the anterior chamber was developed using the ultrasonic image before perfusion, the multi-island genetic algorithm was employed to determine the material parameters of the iris by minimizing the difference between the finite element simulation and the experimental measurements.

**Results:**

Material parameters of the iris in vivo were identified as the iris was taken as a nearly incompressible second-order Ogden solid. Values of the parameters *μ*_1_, *α*_1_, *μ*_2_ and *α*_2_ were 0.0861 ± 0.0080 MPa, 54.2546 ± 12.7180, 0.0754 ± 0.0200 MPa, and 48.0716 ± 15.7796 respectively. The stability of the inverse finite element method was verified, the sensitivity of the model parameters was investigated.

**Conclusion:**

Material properties of the iris in vivo could be determined using the multi-island genetic algorithm coupled with the finite element method based on the experiment.

## Background

Glaucoma is the major cause of irreversible blindness worldwide. Most forms of glaucoma are associated with elevated intraocular pressure(IOP), some forms could lead to abnormalities of the iris morphology. In primary angle closure glaucoma, the iris bows anteriorly, aqueous humor outflow is impeded in moving through the pupil, resulting in intraocular hypertension [1]. In pigment dispersion glaucoma, the iris bows posteriorly, then the pigmented iris epithelium rubs against the lens resulting in pigment liberation, occlusion of the trabecular meshwork [2] and elevated IOP. Therefore, understanding the mechanical properties of the iris may help us to have an insight into the pathophysiology of specific ocular disorders.

As reported in the relevant literature, uniaxial tensile tests are performed on strips and intact tissues of the untreated iris [3], the untreated and stimulated iris by drugs [4]. During inflation tests [5], the highlighted outer contour of the iris is extracted from the cross section image with a laser beam. A nonlinear curve is evinced between the outer surface area change with pressure differential between posterior and anterior chamber. Indentation tests [6,7] were used to determine the mechanical properties on the local regions of the iris. Besides, relative contraction forces of cadaver irides [8] were quantified due to drug stimulation. The mechanical properties of the iris sphincter and dilator muscles were measured by the isometric contraction experiment and the isotonic quick release experiment [9].

Based on the experimental data, investigators used different material models to describe the mechanical behavior of the iris. The linear elastic model [10] was adopted to simulate the inflation test of the ex vivo iris by the finite element (FE) method,but it was only suitable for the lower pressure differential between the posterior and anterior chamber. Somebody [11] tried to use the Neo-Hookean model to simulate the behavior of the iris. However, the simulation results did not completely capture the behavior of the iris. Instead, the Ogden model can be reduced into Neo-Hookean by choosing particular values for α and N [12], and it has been widely employed to determine the mechanical properties of the soft tissues ex vivo [13-18] and in vivo [19-22] such as the cornea [13], blood vessel [14], skin [20] and so on. Besides, the soft tissue was treated as incompressible [23-25].

Results about the mechanical properties of the iris are mostly relied on the ex vivo animal experiments presently. However, mechanical responses of the iris ex vivo are different from that in vivo. For the in vivo experiments it is important to take images on the morphology changes of the iris, advanced equipments could offer high quality anterior chamber images such as the Ultrasonic Biomicroscopy (UBM) and the Anterior Segment Ocular Coherence Tomography (AS-OCT) [26] in clinic. As the UBM and AS-OCT are mainly designed for the human, they are not the optimum choice for animal experiments. Fortunately in vivo studies showed that the Vevo 770 High Resolution Ultrasound System could be used to obtain optimal images over the depth range of the focal region [27], which makes it possible to obtain the images of the iris in vivo.

Therefore, in this study, an in vivo experiment was conducted to obtain the morphology changes of the iris based on the anterior chamber images, the vivo iris was taken as the incompressible Ogden solid, the optimization method was used to determine the material parameters of the iris coupled with the FE method.

## Material and methods

### Animal experiment

#### Experimental animal

Adult New Zealand rabbits (n =6, 2.5-3.5 Kg) without eye diseases were provided by the Experimental Animal Department of the Capital Medical University, animal experiments were conducted in accordance with the National Institute of Health Guide for the Care and Use of Laboratory Animals and approved by the Institutional Animal Care and Use Committee of China.

### Experimental procedure

**Figure 1:**
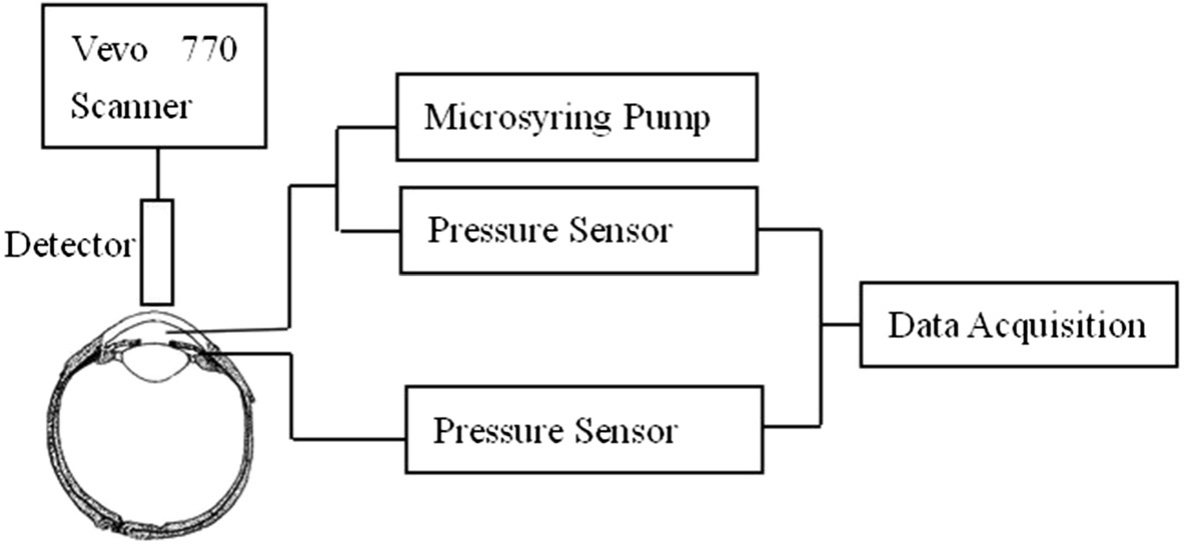
**Schematic diagram of the experimental set-up.** Two 24-G cannulation needles were connected with pressure sensors to record intraocular pressure of the anterior and posterior chamber, respectively. The detector of the ultrasonic system was used to take images of the anterior chamber.

The experimental platform was shown in Figure 1. Before experiments, both pressure sensors were set to a ‘default zero’ calibrated zero pressure. Animals were anesthetized by injection of urethane solution (7.5 ml/kg). Topical anesthetic (Oxybuprocaine Hydrochloride Eye Drops, Santen Pharmaceutical Co., Japan) was also used to avoid eyes’ movement. A 24-gauge needle was inserted from the limbus into the anterior chamber and was connected to a microsyring pump. The other needle was inserted 1-2 mm far from the limbus (near the equatorial sclera) into the posterior chamber. The initial IOP was record when it was stable. The cornea was covered by a layer of ultrasound transmission gel with the transducer positioned in contact with the gel. Before perfusion, reference images of the anterior chamber were taken via Visualsonics Vevo 770 High Resolution Ultrasound System. Then saline was perfused to the anterior chamber at a rate of 100 μL/min by the microsyring pump until the IOP reached 60 mmHg, which took no more than one minute. During the infusion, the IOP was continuously monitored by the pressure sensors and displayed on the screen of the computer in real time, ultrasonic images of anterior chamber were captured at 1 Hz.

### Image processing and analysis

**Figure 2:**
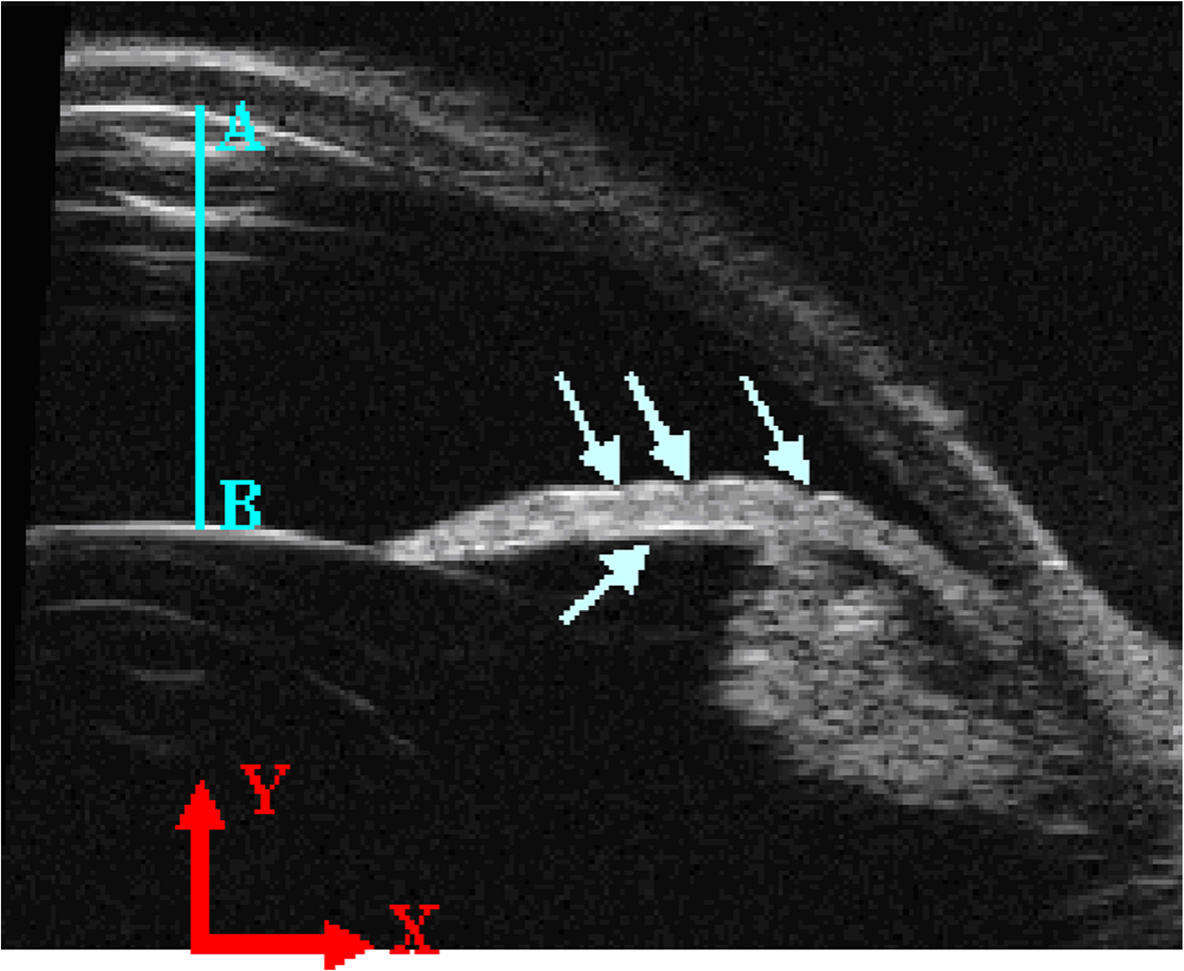
**Ultrasonic image of the anterior chamber.** Arrows point to the characteristic points on the surface of the iris. The anterior chamber depth AB was defined as the distance between two points whose tangent slopes were equal to zero on the edges of the inner surface of the cornea and the upper surface of the lens.

All images were analyzed using the MIMICS software (version 14.12; Materialise) to calculate the anterior chamber depth and displacement changes at different spots of the iris. As shown in Figure 2, the anterior chamber depth AB was defined as the distance between two points whose tangent slopes were equal to zero on the edges of the inner surface of the cornea and the upper surface of the lens. As the surface of the iris is not smooth, there are some characteristic points at the profile edge as shown in Figure 2. The characteristic points were selected in the middle segment and marked by two different independent observers. The displacement was calculated by extracting the coordinate values of the marked point.

The iris root is a ring-shaped narrow band of the periphery of the iris implanted in the ciliary body [28], there is a gap between the iris and lens [29], when the pressure differential increased, the iris will be push backward and the displacement in the X direction is too small to ignore. Therefore, only the displacement in the Y direction was considered.

### Construction of the finite element model

The structure of the anterior chamber is nearly axisymmetric, so the model of the anterior chamber was usually simplified as axisymmetric structure [29-31]. Therefore, we constructed axisymmetric finite element models based on the anterior segment images as follows.

**Figure 3:**
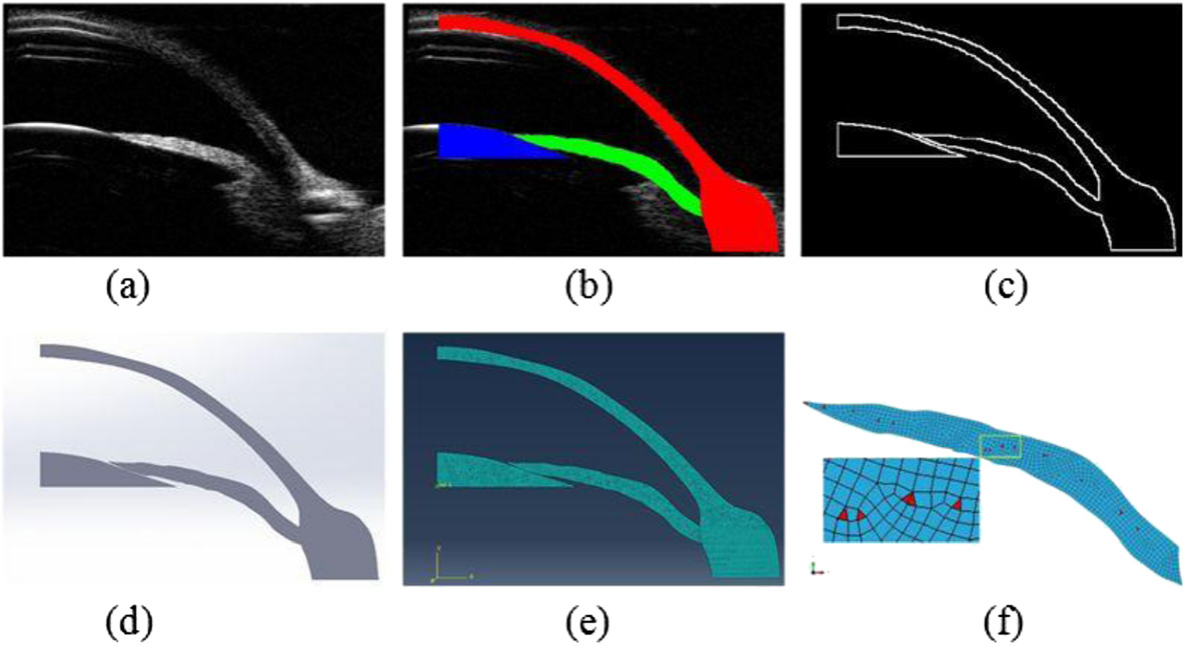
**Construction of the finite element model for the anterior chamber of the iris in vivo before perfusion. (a)** Ultrasonic image of the anterior chamber. **(b)** Cornea, iris and the lens were segmented by optimal threshold segmentation algorithm program and marked in different colors. **(c)** Contours of the anterior chamber extracted. **(d)** Solid model of the anterior chamber. **(e)** A FE model of the anterior chamber based on the solid model. **(f)** The CAX4RH and CAX3 elements were marked in blue and red in finite element model of iris, respectively.

The ultrasonic image perfusion in Figure 3(a) was input to the MIMICS software. Cornea, iris and the lens were segmented by the optimal threshold segmentation algorithm respectively. The lower edge of the iris and the upper surface of the cornea were recognized by local and manual threshold segmentation algorithm. The segmented cornea, iris and the lens were marked in red, green and blue color respectively shown in Figure 3(b). Edges in Figure 3(c) were extracted and translated to the solid model in Figure 3(d). An axisymmetric finite element model of the anterior chamber was constructed based on the solid model as shown in Figure 3(e). The in vivo experiment was simulated by increasing the pressure differential between the anterior and posterior surface of the iris using finite element software ABAQUS (Version 6.12; Simulia), shown in Figure 3(e). Two types of elements were used including CAX4RH and CAX3. CAX4RH is a 4-node bilinear axisymmetric quadrilateral, hybrid, constant pressure, reduced integration, hourglasss control element, and CAX3 is a 3-node linear axisymmetric triangle element, shown in Figure 3(f). The elements of CAX4RH and CAX3 were marked in blue and red, respectively. The elements numbers were summarized for all six finite element models in Table 1.

**Table 1:** Element numbers for all six finite element models

**Specimen**	**Iris**		**Cornea**		**Lens**	
	**CAX4RH**	**CAX3**	**CAX4RH**	**CAX3**	**CAX4RH**	**CAX3**
1	866	25	2514	45	2972	89
2	822	14	2385	40	788	23
3	552	13	2755	69	2681	69
4	799	18	1808	36	572	11
5	685	14	1727	27	2386	66
6	708	14	2129	50	1872	44

The iris was considered as a nearly incompressible Ogden model. The Ogden material model is a hyperelastic material model used to describe the non-linear stress–strain behavior of complex materials such as rubbers, polymers, and biological tissue. The model was developed by Ray W. Ogden in 1972 [32]. The Ogden model, like other hyperelastic material models, assumes that the material behavior can be described by means of a strain energy density function, from which the stress–strain relationships can be derived. These materials can generally be considered to be isotropic, incompressible and strain rate independent. The Ogden model could be expressed by the strain energy function [32,33] as follow:

(1) $$U=\sum_{i=1}^{N}\frac{2\mu_{i}}{\alpha_{i}^{2}}\left({\bar{\lambda }}_{1}^{\alpha_{i}}+{\bar{\lambda }}_{2}^{\alpha_{i}}+{\bar{\lambda }}_{3}^{\alpha_{i}}-3\right)+\sum_{i=1}^{N}\frac{1}{D_{i}}{\left(J_{\mathrm{el}}-1\right)}^{2i}$$

where *U* is the strain energy density per unit volume in the undeformed configuration, *μ*_*i*_, *α*_*i*_ and *D* are material parameters, *J* is the volume ratio, ${\bar{\lambda }}_{i}$ is the deviatoric principal stretch which is related to the principal stretch *λ*_*i*_ by ${\bar{\lambda }}_{i}=J^{-\frac{1}{3}}\lambda_{i}$.

Nowadays, the Ogden model has been widely used to describe the mechanical responses of the soft tissues ex vivo [13-18] and in vivo [19-22] including cornea [13] , artery [14], liver [15], brain tissue [16], placenta tissue [17], spinal cord white matter [18], skin [20], skeletal muscle [22] and so on.

In our study the iris was treated as incompressible second order Ogden model, and the FE model of the anterior chamber is axisymmetric, so D = 1E-6, the material parameters of the in vivo iris are *μ*_1_, *α*_1_, *μ*_2_, and *α*_2_ to be determined.

The cornea was modeled as a linear elastic solid (E = 19.8 MPa, ν = 0.49 [34]).

When the IOP increased in the anterior chamber, the pupillary margin segment of the iris would come into contact with the anterior lens surface, so contact analysis between the lens and the iris was performed with the friction coefficient equal to 0.1. The boundary condition for the limbus was specified by fixing the lower surface. The iris root was tied with the cornea.

**Figure 4:**
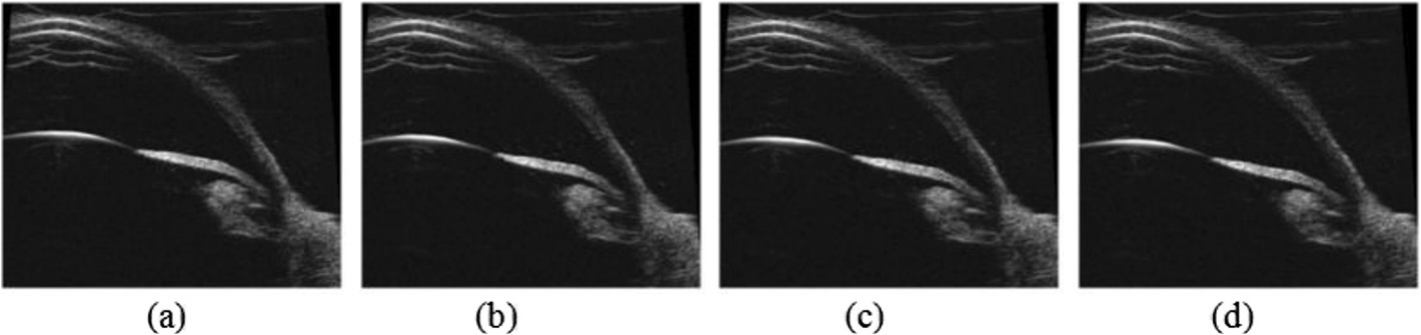
**A serial of images during perfusion with the marked anterior chamber depth.** The IOP increased the anterior chamber was deeper and the lens moved backward. From **(a)-(d)**, the anterior chamber depths were 2.634 mm, 2.681 mm, 2.717 mm and 2.758 mm with the pressure differentials 0 mmHg, 9.12 mmHg, 17.15 mmHg and 25.39 mmHg, respectively between the anterior chamber and posterior chamber.

The lens is located in front of the vitreous body filled with a hydrogel [35] whose modulus of is less than 10 Pa [36,37]. Besides, the lens is connected with the circular zonulas [38,39]. When the pressure differential between the anterior chamber and posterior chamber increased, the circular ciliary zonulas deformed largely which lead to the backward movement of the lens. Therefore, the lens was taken as a rigid body and allowed to move only in the Y direction (Ux = Uz = 0.0). The moving distance of the lens was determined by the anterior chamber depth and the deformation of the cornea. As the apical rise of the cornea varied little when IOP was more than 15 mmHg [40], the moving distance was equal to the change of the anterior chamber depth. In the finite element model, the lens was subjected to the displacement constraints with the same amplitude as the moving distance of the lens at the current IOP level, which could be calculated as the linear relationship [41] between the anterior chamber and IOP was obtained, shown in Figure 4.

Pressure differential between the anterior chamber and posterior chamber was numerically applied to the anterior surface of the iris as a uniform pressure until the pressure differential reached 25 mmHg.

### The inverse method

Material parameters of the iris were identified using an inverse method, where an objective functional was set to quantify the difference between the finite element simulation and the experimental measurements. Material parameters were determined by minimized the value of the objective functional. The objective functional was

(2) $$\epsilon =\frac{1}{N}\sum_{n=1}^{N}{\sum_{p=1}^{P}\left(u^{\text{exp}}-u^{\mathrm{sim}}\right)}^{2}$$

Where *u*^exp^, *u*^*sim*^ are the experimental and model displacements in Y direction, respectively, at surface node n and an IOP level of P, N is the total number of surface nodes and P is the total number of IOP levels.

**Figure 5:**
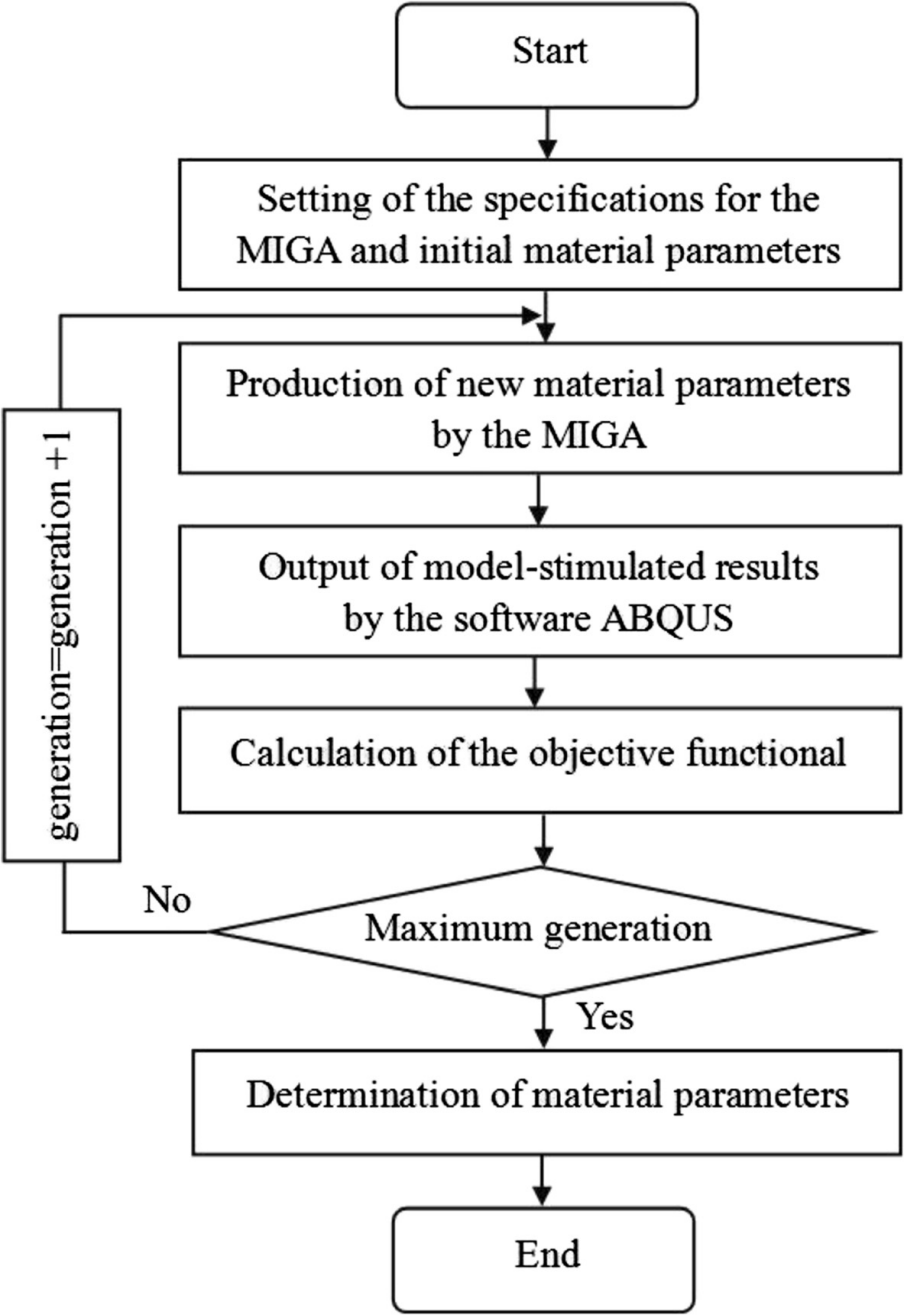
**Flow chart of the inverse method.** Before running, initial parameters were set including the specifications for the MIGA and initial material parameters of the in vivo iris, and the experimental results were input. Then the optimal strategy the MIGA was adopted to produce new material parameters, using the new parameters the software ABQUS calculated model-stimulated results of the displacement in Y direction. According to Formula 2, the value of the objective functional was calculated. Thus one run is over. When the generation reached its maximum number, the optimization would end. Finally the minimum value of the objective functional was got by comparison, responding material parameters were determined which were also called the material parameters of the in vivo iris.

As an optimization algorithm, the multi-island genetic algorithm (MIGA) is a global search optimization search technique. In MIGA each population of individuals is divided into several islands on which all traditional genetic operations are performed separately including selection, crossover and mutation, individuals among the islands can migrate. This approach leads to the Pareto optimum solution in the most efficient manner [42]. The specifications for the MIGA algorithm were shown in Table 2. For one optimization 500 runs were performed.The inverse method was performed as shown in Figure 5, before running, initial parameters were set including the specifications for the MIGA and initial material parameters of the in vivo iris, and the experimental results were input. Then the optimal strategy the MIGA was adopted to produce new material parameters, using the new parameters the software ABQUS calculated model-stimulated results of the displacement in Y direction. According to Formula 2, the value of the objective functional was calculated. Thus one run is over. When the generation reached its maximum number, the optimization would end. Finally the minimum value of the objective functional was got by comparison, responding material parameters were determined which were also called the material parameters of the in vivo iris.

**Table 2:** Multi-island genetic algorithm parameters

**Parameter**	**Value**
Number of islands	2
Number of generations	10
Size of populations	25
Crossover rate	1
Mutation rate	0.01
Migration rate	0.01
Migration interval	5

## Results

**Figure 6:**
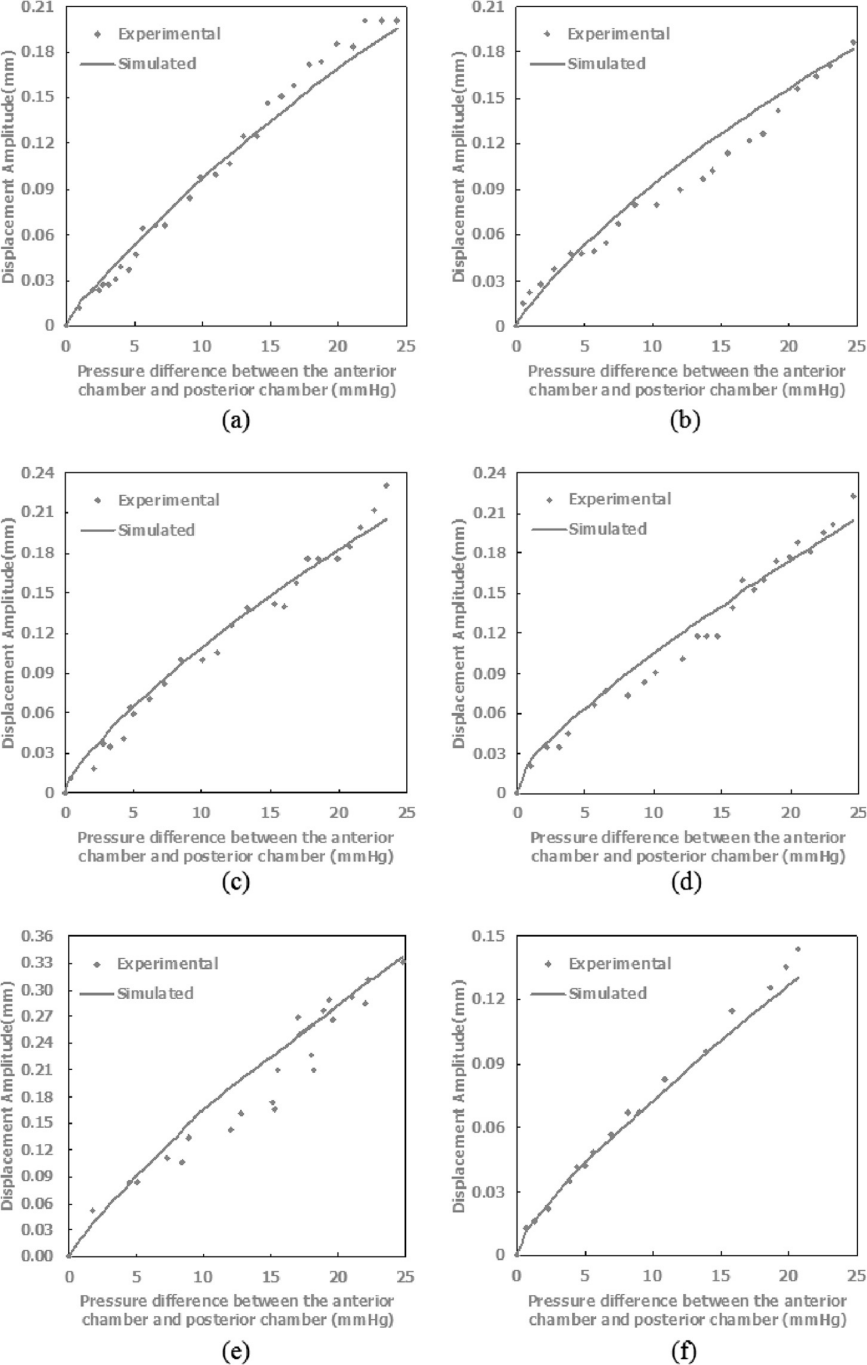
**Comparison of experimentally-measured and model-simulated displacements (in mm) in Y direction for all 6 specimens at one characteristic point. (a)- (f)** Displacement amplitude comparison at one certain point for Specimen 1 to 6.

Material parameters of 6 specimens identified and values of the objective functional were listed in Table 3. For all specimens the objective function values were less than 0.0275 mm^2^. Values of the material parameters *μ*_1_, *α*_1_, *μ*_2_, and *α*_2_ were 0.0861 ± 0.0080 MPa, 54.2546 ± 12.7180, 0.0754 ± 0.0200 MPa, and 48.0716 ± 15.7796 respectively.Figure 6 depicts both experimentally-measured and model-predicted displacement amplitude in Y direction for all 6 specimens. The displacement predicted by the finite element model captured the trend of the experiment’s results well.

**Table 3:** Model parameters results identified using the inverse method

**Set**	***μ***_**1**_**(MPa)**	***α***_**1**_	***μ***_**2**_	***α***_**2**_	***ϵ*** **(mm**^**2**^**)**
1	0.0926	63.2979	0.0976	38.8014	0.0063
2	0.0712	50.3635	0.0444	38.6162	0.0106
3	0.0889	60.0838	0.0725	58.9632	0.0099
4	0.0919	58.3259	0.0972	73.9501	0.0046
5	0.0889	30.1758	0.0671	46.7475	0.0275
6	0.0829	63.2808	0.0735	31.3513	0.0006
Mean	0.0861	54.2546	0.0754	48.0716	
Standard deviation	0.0080	12.7180	0.0200	15.7796	

## Discussion

### Stability analysis

The stability analysis is important for an inverse method because the experiment errors are inevitable. In this paper, specimen 1 was selected following randomization, up to 10% random error was introduced to simulate the experimental error, the materials parameters were identified and compared with the original results. We named the initial parameters as set 1 and three sets of new parameters as set 2 to set 4, shown in Table 4. The results showed most parameters changed less than 8%, only *α*_2_ in set 2 and set 4 varied over 10%. Therefore, the multi-island genetic algorithm coupled with the FE method was feasible to determine the mechanical properties of the iris based on the in vivo experiment.

**Table 4:** Identified parameters for stability analysis

**Set**	***μ***_**1**_**(MPa)**	***α***_**1**_	***μ***_**2**_ **(MPa)**	***α***_**2**_
1	0.0926	63.2979	0.0976	38.8014
2	0.0960	64.3597	0.0951	31.3256
3	0.0987	60.8476	0.0986	41.5219
4	0.0892	66.0751	0.0914	43.9883

### Parameter sensitivity analysis

**Figure 7:**
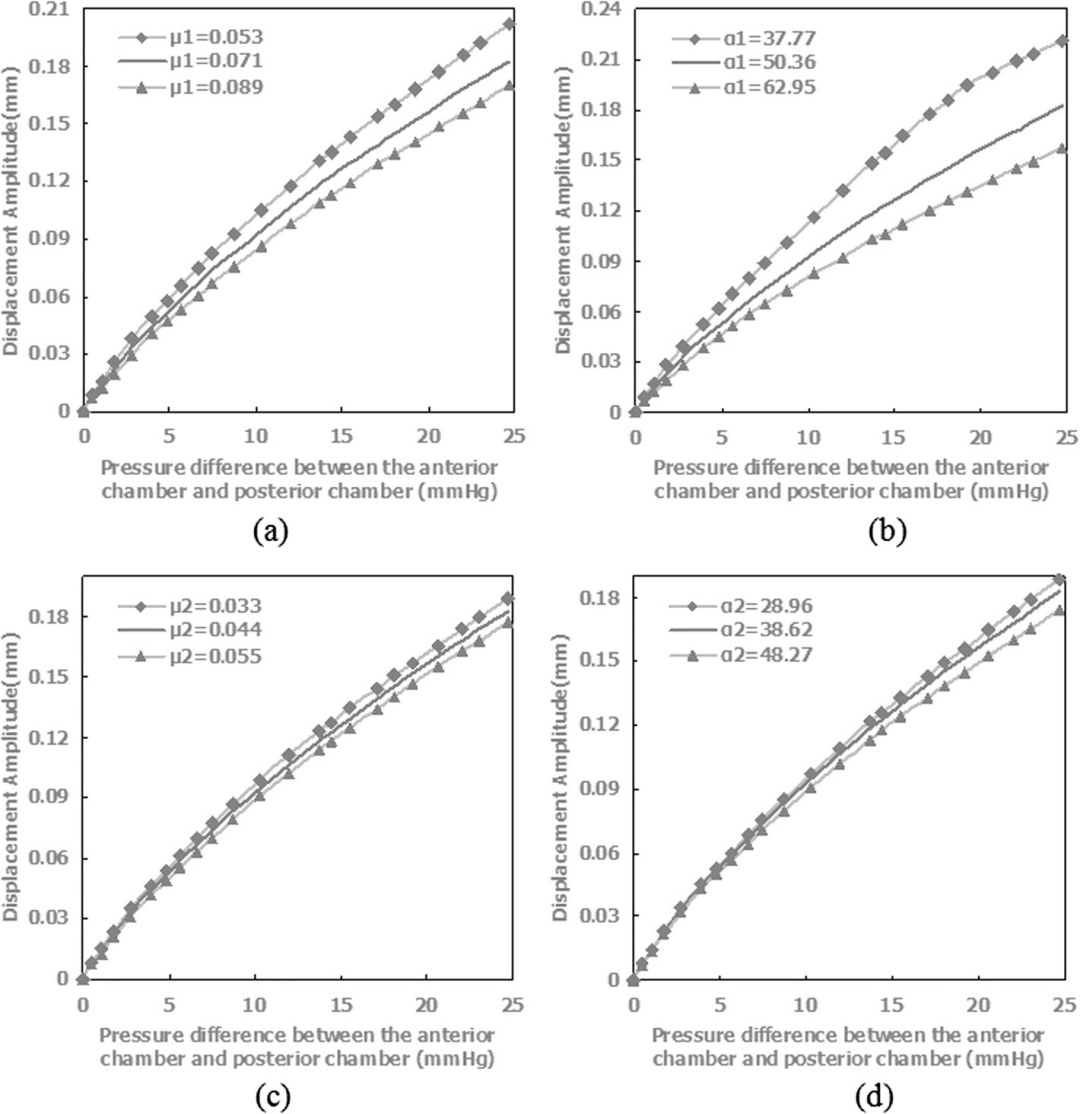
**Displacement amplitude of the characteristic points as a function of pressure differential comparing the effects of a ±25% variation in the material parameters. (a)** the parameter *μ*_1_, **(b)** the parameter *α*_1_, **(c)** the parameter *μ*_2_, **(d)** the parameter *α*_2_.

Parameter sensitivity analysis was performed to study the effect of variations in the materials parameters on the mechanical response. Specimen 2 was chosen to perform parameter sensitivity analysis following randomization. Materials parameters of specimen 2 in Table 3 were used as the baseline. The parameters varied one-by-one by ±25% from the baseline. The results were plotted in Figure 7 for the displacement amplitude in Y direction for one characteristic point. The displacement magnitude increased as the parameters increased. The displacement response was most sensitive to the parameter *α*_1_, more to *μ*_1_ at pressure differentials between the anterior chamber and posterior chamber more than 10 mmHg, less to parameters *α*_2_, and *μ*_2_ whether at higher or lower pressure differentials.

### Limitation

This research had some limitations. There are displacements for the iris in three directions when the pressure differential between the anterior chamber and posterior chamber was applied on the anterior surface of the iris. Considering the iris is an axisymmetric structure, only displacements in two directions are needed to consider. We found displacement in Y direction was more sensitive to the pressure differential than in X direction in the experiment, shown in Figure 4. Therefore, it is reasonable to use the displacements in Y direction to determine the material parameters in our research.

## Conclusion

Acute hypertension could be induced by rapid perfusion to the anterior chamber with the iris morphology changes which could be quantified by image processing and analysis based on the ultrasonic images of the anterior chamber. Mechanical properties of the iris in vivo could be determined using the multi-island genetic algorithm coupled with the finite element method by minimizing the difference between the finite element simulation and the experimental measurements.

In the paper, the iris was considered as an elastic material, in fact the iris is a viscoelastic material [6]. As induction time of the acute ocular hypertension was less than 1 minute in our experiment, the iris showed little viscoelastic response in such a short time, it is valid to consider the iris as a nearly incompressible elastic solid.

In our research the multi-island genetic algorithm coupled with the FE method was verified to determine the mechanical properties of the iris in vivo, individual differences in animals lead to dispersion of the material parameters among the specimens. This method may be used to determine the material parameters for human eyes as medical images by the AS-OCT and UBM could help us construct a realistic model of an individual’s anterior segment. Therefore, obtaining images of the anterior chamber at different IOP levels becomes the prerequisite, provocation tests may be redesigned and developed to induce artificial elevation of the IOP for the future work.

## Abbreviations

IOP: Intraocular pressure; UBM: Ultrasonic biomicroscopy; AS-OCT: Anterior segment ocular coherence tomography; FE: Finite element; MIGA: Multi-island genetic algorithm.

## Competing interests

The authors declare that have no competing interests.

## Authors’ contributions

KZ conducted the in vivo experiments, constructed the finite element model, carried out the optimization work and drafted the manuscript. XQ participated in the numerical simulations, the optimization work and helped to draft the manuscript. MX participated in the animal experiments. CZ conceived of the study design and draft the manuscript. All authors read and approved the final manuscript.
